# Late Failure of Decellularized Cryopreserved Aortic Homograft

**DOI:** 10.1016/j.jaccas.2026.109897

**Published:** 2026-09-02

**Authors:** Fotios Kalafatakis, Uzma Rahman, Sriharsha Talapaneni, Hazar Kadirogullari, Ahmet Kilic

**Affiliations:** a School of Medicine, University of Patras School of Medicine, Patras, Greece; b Division of Cardiac Surgery, The Johns Hopkins Hospital, Baltimore, Maryland, USA; c Georgetown University School of Medicine, Washington DC, USA; d School of Medicine, Koc University School of Medicine, Istanbul, Turkey

**Keywords:** aortic insufficiency, aortic root replacement, decellularized homograft, redo cardiac surgery, structural valve degeneration, SynerGraft

## Abstract

**Background:**

Decellularized aortic homografts were developed to reduce immunogenicity, but very long-term outcomes remain incompletely characterized.

**Case Summary:**

A 54-year-old woman underwent aortic root replacement with a 22-mm SynerGraft decellularized homograft for bicuspid aortic valve disease and aortic root aneurysm. Nineteen years later, she presented with acute heart failure caused by severe aortic insufficiency. Preoperative imaging demonstrated a heavily calcified retrosternal aortic root, guiding a bilateral thoracosternotomy for redo root replacement. Operative and histopathologic findings showed extensive calcific degeneration with left coronary leaflet disruption. Recovery was uneventful.

**Discussion:**

This case illustrates a possible very late failure pattern of a decellularized homograft and underscores the importance of meticulous preoperative imaging and individualized operative planning.

**Take-Home Messages:**

Decellularized aortic homografts may remain susceptible to very late structural degeneration. Careful imaging and tailored surgical planning are essential for complex redo aortic root surgery.

## Visual Summary

**Figure:**
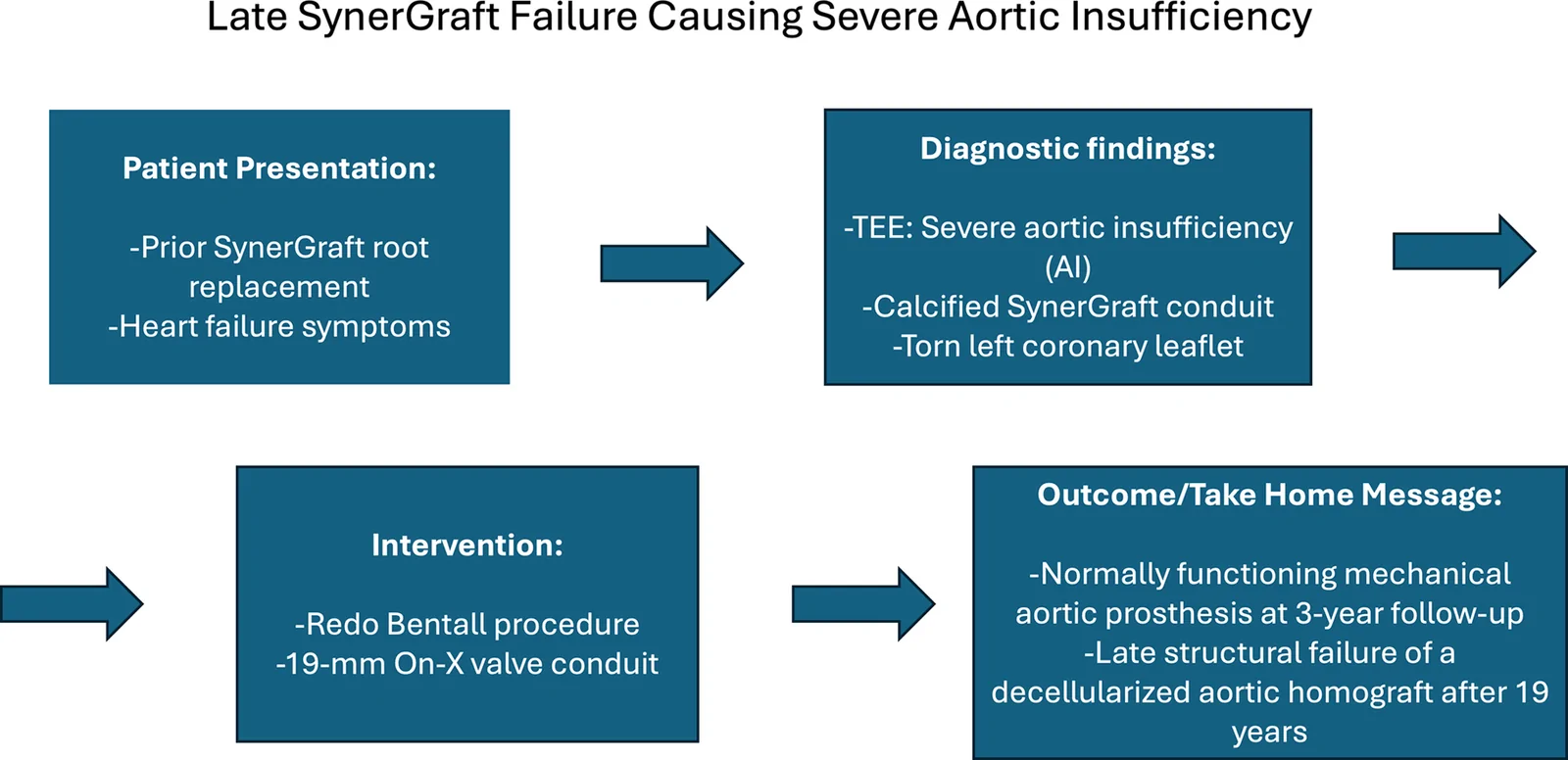

Young patients with combined valvular and aortic root dilatation present a diagnostic dilemma. Root replacement options include a tissue/mechanical Bentall procedure, the Ross procedure, or cryopreserved homografts. For women who prefer to preserve childbearing potential, mechanical conduits are often unsuitable.^1^ Standard cryopreserved homografts are associated with immune-mediated structural degeneration and recipient sensitization, which may complicate future sternotomies or potential heart transplantation.^2^ SynerGraft technology (CryoLife Inc) employs enzymatic decellularization to reduce immunogenicity while preserving the extracellular matrix.^2^, ^3^, ^4^ Although early hemodynamic performance appears comparable to that of conventional cryopreserved grafts, robust long-term durability data in the aortic position remain limited, and challenges related to standardized graft availability and surgical practicality persist.^2^^,^^5^

**Take-Home Messages**

• Decellularized aortic homografts may remain susceptible to very late structural degeneration.
• Careful imaging and tailored surgical planning are essential for complex redo aortic root surgery.

We report a 54-year-old woman presenting with late aortic insufficiency 19 years after SynerGraft root replacement. This case highlights the uncommon observation of prolonged functional durability of a decellularized SynerGraft aortic root followed by a rare mode of very late structural failure characterized by leaflet disruption and acute severe aortic insufficiency. Although a single case cannot establish a causal relationship between decellularization and long-term durability, it provides insight into a potential late-failure phenotype while illustrating both the possible long-term biological performance and the mechanical limitations of decellularized homografts over extended follow-up.

## History of Presentation

A 54-year-old woman presented with acute decompensated heart failure characterized by worsening dyspnea and volume overload.

## Past Medical History

The patient had a history of bicuspid aortic valve stenosis with concomitant aortic root aneurysm. She underwent aortic root replacement with a 22-mm SynerGraft conduit in 2007 because she wished to preserve future childbearing options.

## Differential Diagnosis

The leading diagnosis was structural degeneration of the decellularized aortic homograft causing severe aortic insufficiency secondary to leaflet disruption.

## Investigations

Chest imaging demonstrated bilateral pleural effusions and pulmonary edema, while serum pro-B-type natriuretic peptide was elevated at 4,981 pg/mL. Transthoracic echocardiography demonstrated moderate left ventricular (LV) dilation (LV end-diastolic diameter: 6.0 cm) with preserved systolic function (ejection fraction: 60%-65%), severe left atrial enlargement, and severe aortic insufficiency. Transesophageal echocardiography confirmed severe aortic insufficiency (vena contracta: 0.7 cm), with heavily thickened and calcified homograft leaflets and a mobile echodensity suggestive of leaflet degeneration or perforation. Peak and mean transvalvular gradients were 23 and 12 mm Hg, respectively. Coronary angiography demonstrated no significant coronary artery disease.

Preoperative computed tomography (CT) demonstrated a heavily calcified aortic root/conduit (Figure 1B, Video 1) immediately posterior to the sternum (Figure 1C), raising concern for aortic injury during redo sternotomy.

**Figure 1:**
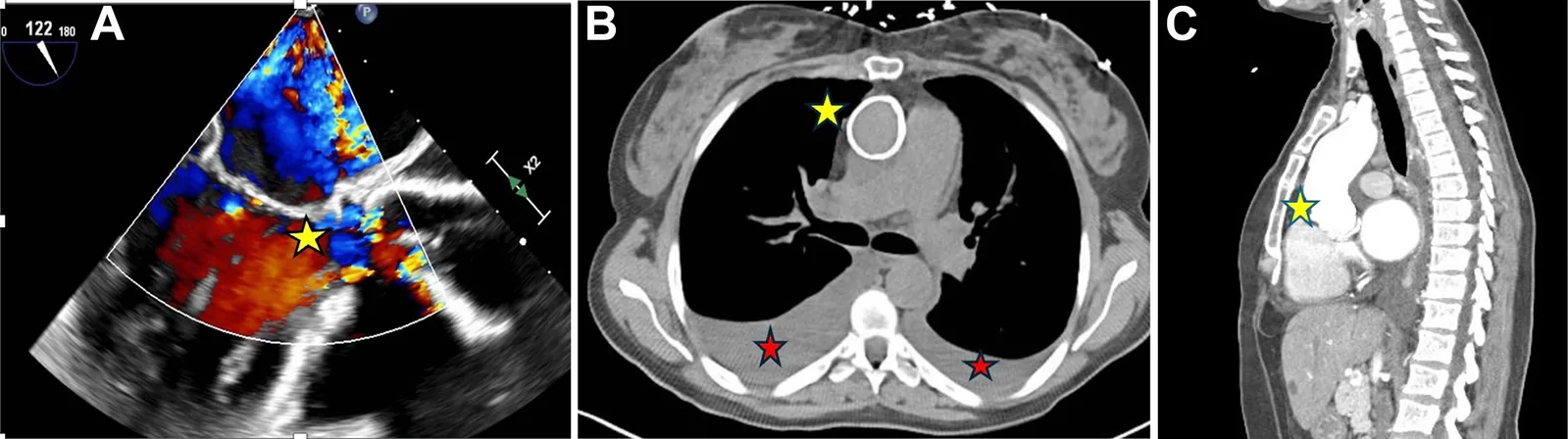
Preoperative Imaging (A) Intraoperative transesophageal echocardiography showing aortic insufficiency in the long axis (star). (B) Computed tomography scan demonstrating extensive calcification of the SynerGraft aortic root/conduit (yellow star) and bilateral pleural effusions (red stars), in axial view. (C) Computed tomography scan showing the proximity of the aorta to the posterior sternum (yellow star), in sagittal view.

## Management

Following multidisciplinary discussion, treatment options, including transcatheter valve intervention, isolated valve replacement, and redo root replacement, were considered. As the patient no longer desired future pregnancy and prioritized durability, a mechanical composite graft was selected.

Intraoperative transesophageal echocardiography confirmed severe aortic insufficiency, consistent with the preoperative diagnosis of leaflet disruption (Figure 1A). Furthermore, given the presence of severe aortic insufficiency, operative planning prioritized access to the LV apex should decompression become necessary during cardiopulmonary bypass initiation. Therefore, the operation was performed through bilateral anterolateral thoracotomies connected by a limited transverse sternotomy (Figure 2A), providing controlled exposure of the retrosternal aorta and direct access to the LV apex. Cardiopulmonary bypass was established using central arterial cannulation in the transverse aortic arch and peripheral venous cannulation via the right common femoral vein to facilitate safe bypass initiation in this complex redo setting. Intraoperative inspection revealed extensive calcification of the SynerGraft conduit and a tear of the left coronary leaflet, explaining the mechanism of severe aortic insufficiency (Figure 2B). Histopathologic examination of the explanted tissue demonstrated calcific degeneration of the aortic conduit and myxoid degeneration with calcification of the aortic valve leaflets, corroborating the intraoperative findings of advanced structural graft deterioration. A Bentall procedure was performed using a 19-mm On-X mechanical valve (On-X Life Technologies Inc).

**Figure 2:**
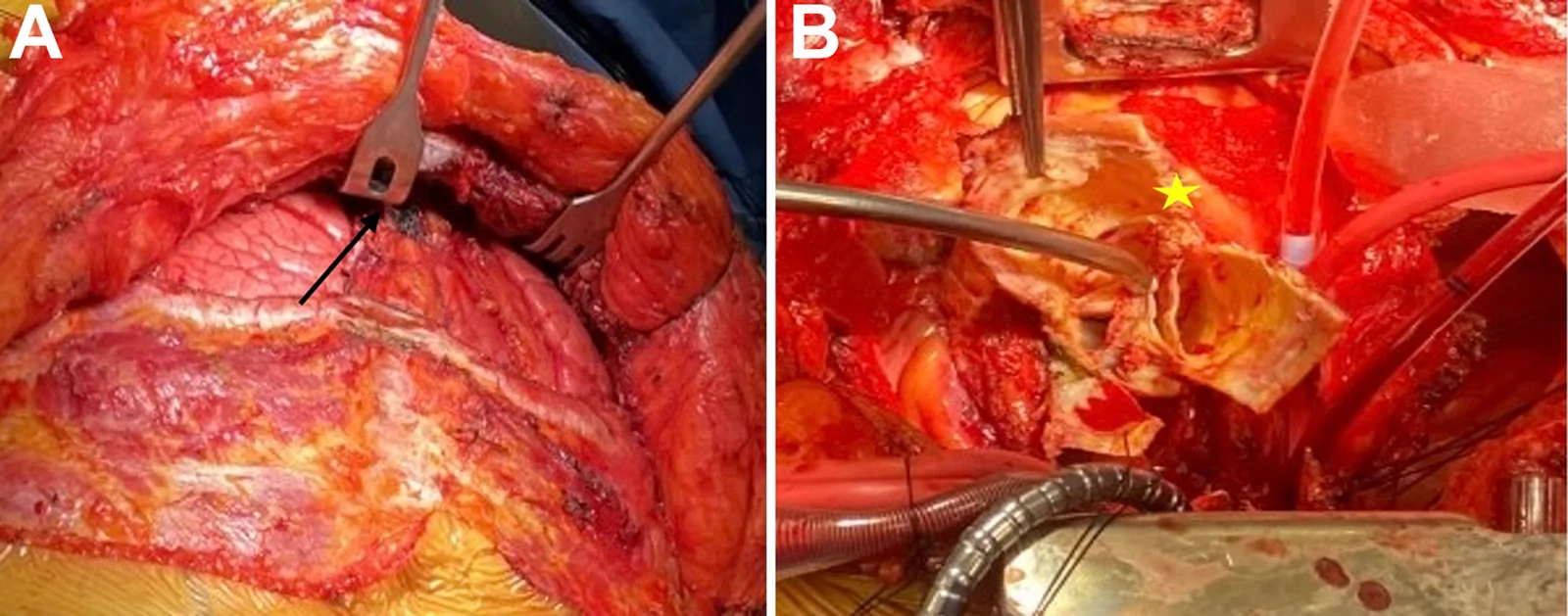
Intra-Operative Findings (A) Intraoperative incision and exposure via thoracosternotomy (black arrow). (B) Leaflet tear and calcification of the SynerGraft aortic root, with disruption of the left coronary leaflet (star), representing the mechanism of severe aortic insufficiency.

## Outcome and Follow-Up

The remainder of the operation and the postoperative course were uneventful. The patient was discharged home on postoperative day 7 on warfarin therapy (goal international normalized ratio: 2-3). At the 3-year follow-up, transthoracic echocardiography demonstrated a normally functioning mechanical aortic prosthesis with preserved leaflet mobility, peak and mean transvalvular gradients of 28 and 18 mm Hg, respectively, and peak velocity of 2.6 m/s. Mild paravalvular regurgitation was present, without recurrent aortic insufficiency or evidence of prosthesis-patient mismatch (Video 2).

## Discussion

This case highlights the prolonged functional durability and the eventual structural limitations observed in a decellularized aortic homograft (DAH). Nineteen years after implantation, the patient developed acute severe aortic insufficiency secondary to leaflet disruption in the setting of extensive calcific degeneration. This clinical course provides insight into a possible late-failure phenotype of decellularized scaffolds, documenting graft performance over nearly 2 decades of follow-up.^6^^,^^7^

The modified Bentall procedure using mechanical composite valve grafts remains the standard for aortic root replacement because of procedural standardization and availability.^7^ However, when evaluating biological alternatives, decellularized aortic homografts may offer potential immunologic advantages over conventional cryopreserved homografts. Standard cryopreserved homograft typically elicits a broad and persistent humoral response to donor human leukocyte antigens, leading to elevated panel reactive antibody levels, early tissue calcification, and structural valve degeneration.^2^, ^3^, ^4^ By using enzymatic decellularization to provide a nonreactive scaffold for potential autogenous remodeling, DAH aims to mitigate this immune-mediated injury and reduce the calcific degeneration frequently observed with conventional cellularized homografts.^3^^,^^4^^,^^8^ Current clinical evidence supporting regenerative heart valves has expanded through studies such as the ESPOIR (European Clinical Study for the Application of Regenerative Heart Valves) trial, which has contributed to the growing clinical experience with decellularized tissue-engineered homografts.^9^ Nevertheless, comparative durability data for DAH in the aortic position remain limited, and much of the available long-term evidence is derived from the pulmonary position and the Ross procedure.^3^^,^^7^^,^^8^ In the pulmonary position, nearly a decade of clinical experience with fresh decellularized homografts supports their safety and functional performance, with observational studies reporting improved 10-year freedom from conduit dysfunction and reintervention compared with conventional cellularized homografts.^4^^,^^9^ Although these findings are encouraging, the late structural failure observed in our patient nearly 2 decades after implantation suggests that prolonged durability does not preclude eventual structural degeneration and that long-term outcomes likely reflect a complex interaction between biologic and mechanical factors.^10^

The mechanism of failure in this case was likely multifactorial, characterizing a rare late-failure phenotype of decellularized scaffolds. Intraoperative inspection and histopathologic evaluation demonstrated extensive calcific degeneration of the conduit and myxoid degeneration with calcification of the valve leaflets, suggesting that progressive leaflet stiffening increased susceptibility to chronic cyclic stress and eventual tearing.^6^^,^^8^ While SynerGraft technology significantly reduces antigenic burden to mitigate early immune injury, the contribution of residual biologic degeneration cannot be completely excluded.^10^ Evidence suggests that residual immunogenicity in decellularized matrices can elicit highly individual recipient responses, potentially leading to the late structural fatigue observed nearly 2 decades postimplantation.^10^ Despite this eventual failure, the prolonged durability observed in this patient is consistent with the proposed immunologic benefits of decellularization; however, no causal relationship can be established from a single case. Standard cryopreserved homografts often trigger elevated panel-reactive antibody levels, which can complicate future sternotomies or restrict the donor pool for younger patients, who may eventually require heart transplantation.^2^^,^^3^ In the present case, structural valve function was maintained for 19 years before failure occurred. Although this prolonged durability is noteworthy, it should be interpreted cautiously and cannot be attributed solely to the decellularized nature of the graft.^7^^,^^8^

This case highlights the importance of meticulous preoperative CT planning in patients undergoing complex redo aortic root surgery. Reoperative cardiac surgery is inherently more challenging than primary surgery because of adhesions and the increased risk of injury to mediastinal and cardiovascular structures during sternal re-entry. Recent experience has shown that a structured CT-based preoperative planning strategy—including assessment of sternal re-entry, cannulation, and the planned operative approach—may facilitate operative planning and has been associated with shorter operative and cross-clamp times as well as reduced hospital length of stay. Furthermore, detailed preoperative cross-sectional imaging has been associated with fewer perioperative complications while providing a comprehensive anatomical roadmap that may improve operative planning and team preparedness.^9^ In our patient, these findings supported the decision to perform a bilateral thoracosternotomy, allowing controlled exposure of the retrosternal aorta and safe establishment of cardiopulmonary bypass.

## Conclusions

Beyond operative planning, continued advances in biologic conduit development remain important. The development of manufacturing processes independent of human donor tissue may ultimately improve the availability of these biologic conduits for complex aortic disease management.^7^

**Visual Summary:**
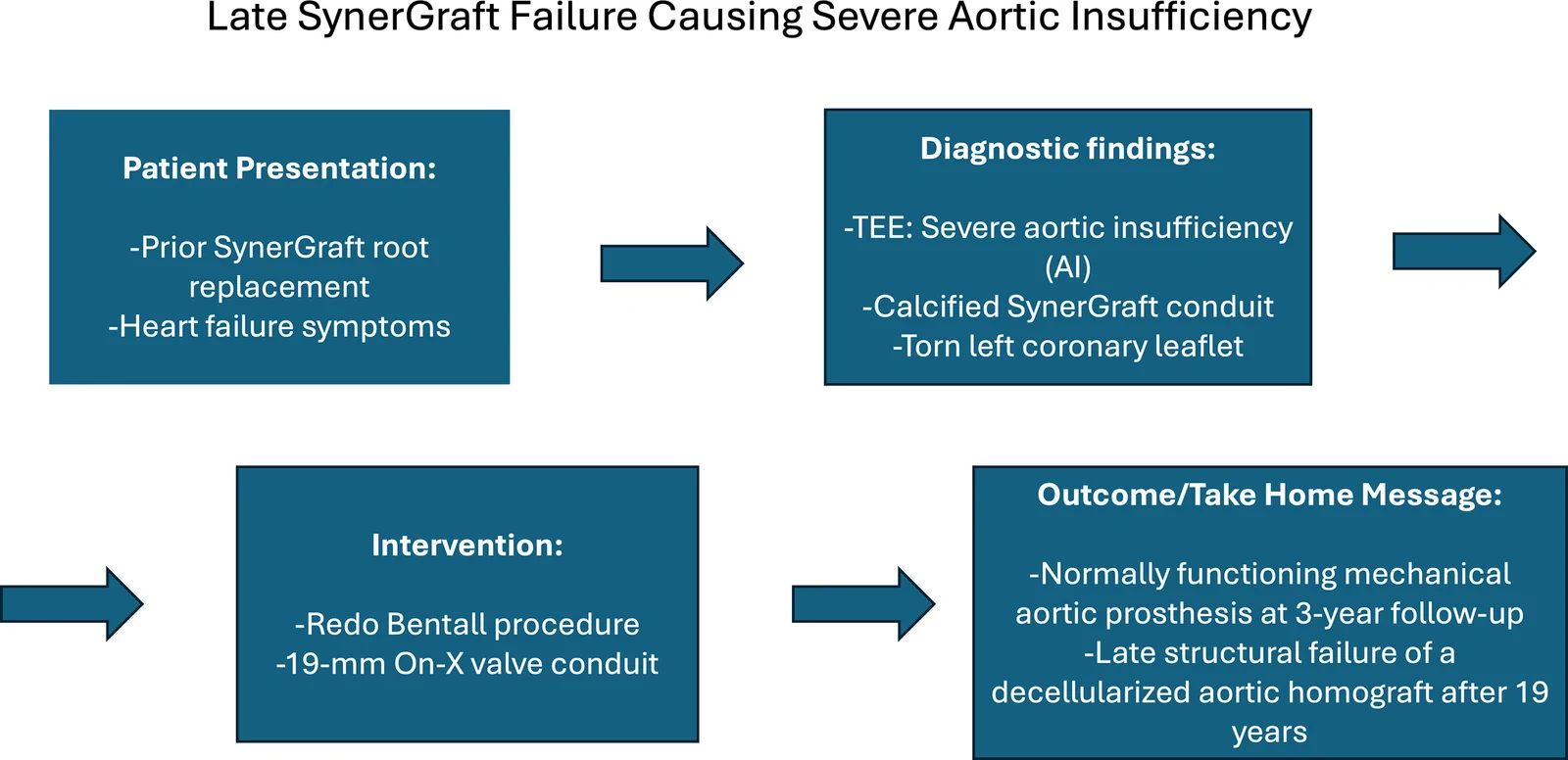
Clinical Overview of a Patient Presenting With Late Structural Failure of a Decellularized SynerGraft Aortic Homograft 19 Years After Implantation The visual summary illustrates the patient's presentation with acute severe aortic insufficiency, the key diagnostic findings, the operative management with redo Bentall root replacement, and the favorable clinical outcome at 3-year follow-up, highlighting the importance of meticulous preoperative computed tomographic planning in complex redo aortic root surgery. TEE = transesophageal echocardiography.

### Ethics Statement

Institutional Review Board approval was not required for this single-patient case report at the Johns Hopkins Hospital. Oral consent for publication of anonymized data was obtained from the patient.

## Funding Support and Author Disclosures

The authors have reported that they have no relationships relevant to the contents of this paper to disclose.

## Appendix

Video 1 Preoperative Computed Tomography Reconstruction Demonstrating Extensive Calcification of the SynerGraft Aortic Root/Conduit and Valve Leaflets, Consistent With Advanced Structural Degeneration Identified Intraoperatively and on Histopathologic Evaluation

Video 2 Postoperative Transthoracic Echocardiography at 3-Year Follow-Up Demonstrating Preserved Mobility of the Mechanical Aortic Valve Prosthesis With Satisfactory Hemodynamic Performance and No Recurrent Aortic Insufficiency
